# Supplementary material for: Introducing a blocked procedure in nonparametric CD-CAT
Source: PLoS One. 2024 Dec 10;19(12):e0312747. doi: 10.1371/journal.pone.0312747 (PMC11630612; doi:10.1371/journal.pone.0312747)
Supplement: Attachment — Submitted filename: Response to reviewers R2.docx [file pone.0312747.s002.docx]

Dear Editor,

We sincerely appreciate the time and effort you and the reviewers have invested in evaluating our manuscript. We have carefully considered all the valuable comments and suggestions provided, and have made comprehensive revisions to the paper. Below is a summary of the major modifications:

- We have enhanced the introduction by including relevant literature on nonparametric methods, as recommended by the editor.
- Detailed steps and the rationale behind the proposed procedures have been added.
- The Methods section has been expanded to clarify the rationale for the simulation plan.
- The tables in the Results section have been reorganized to improve clarity.
- The Discussion section has been strengthened to address the reviewers’ concerns more thoroughly.
- We have conducted a comprehensive grammar check throughout the manuscript. All identified grammatical errors have been corrected to ensure that the text is clear, concise, and professional.

We would like to thank you for your question regarding the construction of item banks in non-parametric CD-CAT. We appreciate the opportunity to clarify this aspect of our study.

Compared to parametric approaches, non-parametric CD-CAT indeed places relatively lower demands on item quality. In non-parametric methods, the reliance on strict model assumptions is reduced, which allows for greater flexibility in item selection. As a result, non-parametric methods can effectively function even when items exhibit lower levels of discrimination.

However, it is important to emphasize that the fundamental criteria of item quality remain crucial, regardless of whether a parametric or non-parametric model is used. For example, key aspects such as content validity and the clarity of item wording are universally applicable. To construct a high-quality item bank without relying on a specific measurement model, we suggest focusing on general principles of test development. This includes ensuring that the items comprehensively cover the targeted skills or attributes and that they are of appropriate difficulty for the population being tested. Furthermore, empirical analyses, such as item-total correlations, can be employed to identify and remove poorly functioning items. These steps help in maintaining the overall quality of the item bank, even in the absence of model-based item calibration.

In response to your comment, we have revised and supplemented the relevant section of the Discussion in our manuscript (see pp. 36-37). We have expanded our discussion on item bank construction, addressing the specific considerations for non-parametric CD-CAT. This includes the challenges of maintaining item quality and the strategies for constructing a robust item bank without relying on parametric models.

We hope that this response and the corresponding revisions to the manuscript address your concerns effectively.

Please find attached our detailed responses to the reviewers' comments, along with an outline of the revisions made. All modiﬁcations are highlighted in the revised manuscript. We greatly appreciate your continued consideration of our work and the constructive feedback provided.

Sincerely,

Authors

**Response to Reviewer 1**

We appreciate the time and eﬀort that you have devoted to giving us insightful suggestions and comments. We thoroughly revised our paper according to the suggestions and comments from you and the other reviewer.

Below, we have arranged our responses below in the same sequence as your original comments/suggestions and outline changes made to the paper according to your comments/suggestions. All modiﬁcations are highlighted in the revised manuscript.

1. **The authors explored two versions of each blocked procedure in the simulation study, can the authors explain the reasons?**

**Reply:**

Our decision to explore multiple versions of the blocked procedure is grounded in the work of Kaplan and de la Torre (2020), who proposed different versions of the blocked parametric CD-CAT, including unconstrained, constrained, and hybrid approaches.

We provided a detailed explanation of the rationale behind the constrained version in the revised manuscript (pp. 13-14):

*“The constrained version of the blocked procedure aims to make the q-vectors of items within the same block as distinct as possible. The rationale behind introducing the constrained version lies in its potential improvement on the performance of the adaptive testing procedure. Previous research in parametric methods indicated that administering items with the same q-vector repeatedly did not provide additional information for improving measurement precision [29]. Kaplan and de la Torre introduced the constrained version to their parametric blocked procedure to enhance the diagnostic power of the test by avoiding redundancy and ensuring a broader coverage of the attribute space [11]. ”*

2. **Can the authors list the detailed steps of the blocked NPS and GNPS methods in the main text?**

**Reply:**

"**The proposed blocked procedures**" section has been reorganized and largely rewritten to enhance clarity, with a detailed description of the steps involved in the blocked NPS and GNPS now provided in the main text. Furthermore, **Fig 1** has been incorporated to visually represent the unconstrained and constrained versions of the blocked nonparametric CD-CAT procedure.

3. **“how to determine the number of items that each q-vector type needed in the first step for the constrained blocked GNPS (lines 208-210)”**

**Reply:**

We apologize for the confusion caused by the original wording. This section has been revised to improve clarity. Since the number of q-vector types is generally larger than the block size, $J_{s}$ q-vector types are randomly selected in the constrained blocked GNPS. Each chosen q-vector type contributes one item to form the block, meaning that the number of items that each q-vector type needed is either one or zero. The revised manuscript now reads,

“*In the constrained version of the blocked GNPS, since the number of distinct q-vector types M (e.g., M=7 when examining three independent attributes) typically exceeds the block size* $J_{s}$*, only one item from each q-vector type is required. The items with the highest discrimination power (defined in Equation 3) within each q-vector type are identified, and the top* $J_{s}$ *items from this subset are selected to form the block.*” (p. 14)

4. **The reason of the simulation design should be explained.**

**Reply:**

We have rewritten the Method section to provide explanation of the simulation design in the revised manuscript on pages 16-19.

5. **During the data generation, only six attribute profiles were generated, the rests were discarded, the authors should explain this setting.**

**Reply:**

We regret any confusion caused by the original writing. This section "Examinee generation" has been revised to improve clarity (pp. 21-22).

The decision to focus on a subset of six attribute profiles is based on the methodology outlined by Kaplan et al. (2015), who employed a similar approach to design a more efficient simulation study. The six selected attribute profiles—ranging from no mastery to full mastery—represent key stages in attribute mastery: $\boldsymbol{\alpha}_{\boldsymbol{0}}=\left( 0,0,0,0,0 \right)$, $\boldsymbol{\alpha}_{\boldsymbol{1}}=\left( 1,0,0,0,0 \right)$，$\boldsymbol{\alpha}_{\boldsymbol{2}}=\left( 1,1,0,0,0 \right)$，$\boldsymbol{\alpha}_{\boldsymbol{3}}=\left( 1,1,1,0,0 \right)$，$\boldsymbol{\alpha}_{\boldsymbol{4}}=\left( 1,1,1,1,0 \right)$，and $\boldsymbol{\alpha}_{\mathbf{5}}=\left( 1,1,1,1,1 \right)$.

Since the attributes are assumed to be independent in our study, all attribute mastery patterns involving the same number of mastered attributes can be considered equivalent in terms of their simulation outcomes. By selecting one representative profile for each level of mastery (i.e., the number of attributes mastered), we can capture the essential dynamics of the attribute space without generating and analyzing all possible profiles.

This more efficient simulation design allows us to significantly reduce computational time and resources while maintaining the generalizability of our findings.

6. **The authors should explain the purpose and reason of constraining the q-vector of blocked items. Did this situation (i.e., only those items with different q-vectors would be selected into the block) would be occurred in empirical study?**

**Reply:**

This is related to the first comment. Additionally, we have strengthened the discussion regarding the empirical study：

“*The constrained version of the blocked procedure is also worth considering, as it can maintain or even enhance classification accuracy under certain conditions by constraining the q-vectors of blocked items. Since items with identical q-vectors assess the same content or skills, incorporating a variety of item types within a block is beneficial to avoid redundancy and reduce examinee boredom*.”(p. 36)

7. **During the simulation two (lines 375-385), which methods (package(s)) were used to calibrate item parameters? The original EM algorithm? or the Bayes Modal Estimation method (Ma & Jiang, 2021). Meanwhile, how many people were used to calibrate the items?**

**Ma, W., & Jiang, Z. (2021). Estimating cognitive diagnosis models in small samples: Bayes modal estimation and monotonic constraints. Applied Psychological Measurement, 45(2), 95-111.**

**Reply:**

We acknowledge the reference provided (Ma & Jiang, 2021). This new method enhances estimation accuracy, particularly when dealing with small sample sizes (≤100). As the sample size and test length increase, the differences between the new method and other methods become less significant. Since our focus is not on improving the calibration accuracy of parametric methods, we chose the conventional EM algorithm for simplicity. For our purpose, the use of parametric methods with small calibration samples was included to ensure a comprehensive comparison to underline the advantage of the non-parametric method over the parametric method with substantial calibration errors.

We have clarified in the revised manuscript:

“*To demonstrate the impact of small calibration samples on the performance of the parametric methods, the calibration sample size (*$N_{0}$*) was set to 100 [8,10]*.” (p. 22)

“*The R package GDINA was used for item calibration in the parametric item selection strategies, specifically for the DINA and A-CDM models [32], using the marginal maximum likelihood estimation (MMLE) method with the Expectation-Maximization (EM) algorithm.*” (p. 20)

We have strengthened the discussion to inform readers of the existence of new method that better calibrate items in small sample size scenarios:

“*First, the nonparametric approach was introduced to address challenges associated with parametric methods, particularly in scenarios involving small calibration samples. However, recent advancements in parametric methods have yielded techniques that enhance estimation accuracy, even with small sample sizes (≤100) [36]. Future research should incorporate these developments when comparing nonparametric and parametric approaches.*” (pp. 37-38)

8. **During the calibration of item parameters, how to calibrate the item parameters? all 1240 items were assembled to create a single test, then calibrated using small samples (e.g., 100)? Is that realistic? Can the authors adopted the matrix sampling technique to calibrate the item parameters?**

**Reply:**

Thank you for your insightful comment. As this is a simulation study, it is indeed feasible to administer all 1,240 items to a calibration sample of 100 examinees during the calibration of item parameters. While this approach would be highly unrealistic in a real-world setting due to practical constraints, it is appropriate within the context of a simulation study, where the goal is to showcase the impact of calibration sample sizes on the parametric methods. In real-world applications, a matrix sampling technique would indeed be a more practical and realistic option for calibrating item parameters. However, the exploration of such techniques is beyond the scope of the current study, which is focused on the theoretical aspects of the proposed method.

9. **Can the authors explain why the classification accuracy rate decreased as the block size increased?**

**Reply:**

Thank you for highlighting this important issue. We have accordingly expanded and strengthened the relevant section of the discussion.

It is important to note that a block size of 1 corresponds to the traditional item-level CD-CAT administration, where the test is highly adaptive, selecting the most informative item for each examinee at each stage. However, as the block size increases, the test becomes less adaptive, akin to the difference between Multi-Stage Testing (MST) and item-level CAT. In a blocked CD-CAT, multiple items are selected at once, reducing the ability to tailor each item to the examinee’s evolving ability estimate.

This reduced adaptivity is a key reason why classification accuracy tends to decrease as block size increases. Kaplan and de la Torre (2020) observed similar findings, particularly under conditions where items were of low quality and the test lengths were short or medium.

Ultimately, the choice of block size involves a trade-off between adaptivity and other practical considerations, such as allowing students to review and change their answers. As Kaplan and de la Torre (2020) suggest, practitioners must determine the tolerable level of loss in classification accuracy when deciding on the appropriate block size for their specific context.

We have expanded our discussion on the decreased accuracy with increased block size (see p. 34-35).

10. **The authors information were missing for the 18th reference in the "references" section**

**Reply:**

Thank you for pointing out this. We apologize for this oversight and will ensure that the correct author details are included in the revised manuscript.

Yan D, Von Davier AA, Lewis C. Computerized multistage testing: Theory and applications. New York: CRC Press; 2014.<https://doi.org/10.1201/b16858>

We are grateful for your careful attention to detail and the chance to refine our manuscript. We believe these revisions have greatly enhanced its clarity and coherence. Thank you again for your insightful feedback. We are confident that the manuscript is now stronger and more suitable for publication.

**Response to Reviewer 2**

We appreciate the time and eﬀort that you have devoted to giving us insightful suggestions and comments. We thoroughly revised our paper according to the suggestions and comments from you and the other reviewer.

Below, we have arranged our responses below in the same sequence as your original comments/suggestions and outline changes made to the paper according to your comments/suggestions. All modiﬁcations are highlighted in the revised manuscript.

1. **The simulation design used a parametric CD-CAT design, with some uncommon settings. Can the authors explain on the rationale for doing such a design, as well as the relationship to a non-parametric CD-CAT and more explanations of the simulation design? The explanations and specifications can assist the reader in understanding the distinction between non-parametric and parametric CD-CATs, conducting a non-parametric CD-CAT with proper simulation settings, and drawing the simulation results or implications. Such as, a big item pool (1240 items) is employed, which is rare in small-scale or classroom contexts. Was the sample size of 200 subjects for each studied attribute mastery pattern large enough to make an inference? Why were just the DNIA and A-CDM models used? The rationale for setting up the three levels of test lengths, etc.**
   a) **The simulation design used a parametric CD-CAT design, with some uncommon settings. Can the authors explain on the rationale for doing such a design, as well as the relationship to a non-parametric CD-CAT and more explanations of the simulation design?**

**Reply:**

This important point resonates with the fourth comment from the first reviewer. We have provided an explanation of the simulation design in the revised manuscript on pages 16-19.

b) **big item pool (1240 items) is employed, which is rare in small-scale or classroom contexts.**

**Reply:**

Thanks for bringing this out. The use of a large item pool (1240 items) in our simulation study was intentional to isolate the effects of the proposed method from those of item pool size. While we recognize that such a large item pool may be uncommon in small-scale or classroom contexts, the goal was to avoid confounding the results with the limitations that a smaller item pool could introduce. Our method is designed to work with smaller item pools as well; however, in scenarios where the item pool is limited and does not provide adequate items to meet the needs of the assessment, the performance of the method could be constrained. Therefore, the large item pool in our study serves to ensure that the method’s efficacy is assessed independently of the item pool’s size, allowing for a clearer evaluation of its potential under optimal conditions.

We explained the choice of the sample size in the "Method" section: "*The large item pool serves to ensure that the proposed method’s efficacy is assessed independently of the item pool’s size, allowing for a clearer evaluation of its potential under optimal conditions.*" (p. 21)

We discussed future directions related with the sample size in the "Discussion" section: "*Second, our simulation study focused on evaluating the proposed method using a large item pool to minimize the confounding effects of pool size. However, since large item pools may be impractical in real-world settings, future research should assess its performance with smaller pools and determine the minimum pool size required.*" (p. 38)

c) **Was the sample size of 200 subjects for each studied attribute mastery pattern large enough to make an inference?**

**Reply:**

Thank you for your insightful question regarding the sample size. In our study, we selected a sample size of 200 subjects per attribute mastery pattern, informed by prior research and simulation studies in the field (Chang et al., 2019; Chiu & Chang, 2021). Previous research has shown that an overall sample size of 500 or 1,000 subjects, encompassing 31 attribute profiles, is sufficient to achieve stable and reliable parameter estimates when dealing with five independent attributes. This overall sample size implies a significantly smaller number of subjects per attribute profile than the 200 we employed.

Chang, Y. P., Chiu, C. Y., & Tsai, R. C. (2019). Nonparametric CAT for CD in Educational Settings With Small Samples. *Applied Psychological Measurement*, *43*(7), 543–561. <https://doi.org/10.1177/0146621618813113>

Chiu, C.-Y., & Chang, Y.-P. (2021). Advances in CD-CAT: The General Nonparametric Item Selection Method. *Psychometrika*, *86*(4), 1039–1057. <https://doi.org/10.1007/s11336-021-09792-z>

d) **Why were just the DNIA and A-CDM models used?**

**Reply:**

We have explained the model choice in the revised manuscript, which now reads

“*The DINA model and the additive cognitive diagnosis model (A-CDM) were used for data generation and parametric CD-CAT simulations, as their underlying assumptions align well with cognitive processes commonly observed in academic contexts, such as mathematics education [30]. The DINA model assumes a conjunctive relationship between attributes, consistent with the assumptions of the NPS method. In contrast, the A-CDM adopts a compensatory relationship between attributes, which is compatible with the GNPS method [7-10,30].*” (p. 18)

e) **The rationale for setting up the three levels of test lengths,**

**Reply:**

We have explained the choice of test lengths in the revised manuscript, which now reads

“*At the initial stage, five items were selected using the Q-optimal algorithm [31], after which the blocked design was implemented. Test lengths of 17, 25, and 33 items were used to represent short, medium, and long tests, respectively. A test length of 17 implies that 12 additional items were administered after the initial five, corresponding to three blocks when the block size is four. These test lengths exceed those typically employed in parametric blocked designs [11], as nonparametric methods generally require longer tests to achieve comparable efficiency.*” (pp. 18-19)

2. **The authors recommended that the blocked NPS is more suited for the DINA model and the blocked GNPS for the A-CDM model. The empirical data may be collected from different types of CDMs; could the authors provide further explanations / advices on when and how to utilize a suitable item selection method for a non-parametric CD-CAT when various CDMs are used. For example, when using a non-parametric CD-CAT with the DINO model, which item selection strategy should be used?**

**Reply:**

We appreciate this insightful comment. You are correct that empirical data may arise from different types of CDMs beyond DINA and A-CDM.

For models like DINO, which assumes a disjunctive relationship between attributes, the blocked NPS method can be applied but the ideal response is calculated differently than in the case of conjunctive relationships. We have added the relevant information in "The proposed blocked procedure".

In response to your suggestion, we've also expanded our discussion on the limitations and future directions, which now reads,

"*Third, the simulation study focused on data generated from a single model (either DINA or A-CDM). Future research should explore the performance of the proposed procedures in item pools where various CDMs are mixed. Based on previous findings with item-level CD-CAT using GNPS [10], we anticipate that the blocked GNPS method would outperform the blocked NPS in such scenarios.*" (p. 38)

3. **The results of the non-parametric CD-CAT were not included in Table 3.**

**Reply:**

Thank you for your attention to the details in our manuscript. We have clarified that “Parametric methods using inaccurately estimated item parameters were only paired with the DINA model for data generation model, as this was not the primary focus of the present study. ” Upon revising our manuscript, we have combined the original Table 2 and Table 3 to streamline the presentation of our data. The non-parametric CD-CAT results, which are consistent between the two tables, have been included in the newly merged table.

4. **On pages 24 and 29, the authors mentioned that “The violation of NPS's underlying assumptions when generating response data from the A-CDM model resulted in relatively low overall pattern classification accuracy for NPS.” and “When the response data better conform to the A-CDM model assumptions, using GNPS is more effective.” It is hard to follow if a reader is not familiar with NPS/GNPS and A-CDM.**

**Reply:**

We appreciate your feedback and understand that the explanation could be challenging for readers unfamiliar with NPS/GNPS and A-CDM. To make our findings more accessible, we added description about the relationship between NPS and DINA and the relationship between GNPS and A-CDM (p. 18) in the "Simulation design" section.

The original writing in the Results and Discussion sections has also been improved to increase clarity:

“*Table 3 shows the performances of the item selection strategies when the response data were generated using the A-CDM, which assumes a compensatory relationship between attributes. We observed relatively low overall pattern classification accuracy for the NPS method, which assumes a conjunctive relationship between attributes. Overall, the effects of item quality, test length, and block size on pattern classification accuracy were generally comparable to those observed when response data were generated from the DINA model, which shares the same assumption as the NPS method.*” (p. 29)

“*Conversely, if the attributes exhibit a more complex relationship, as in the case of the A-CDM, the blocked GNPS procedure is more suitable. Importantly, the exact CDM need not be known, as the weights in the GNPS method automatically adjust to the complexity and variability of the response data [9,10].*” (pp 35-36)

5. **Lines 451-454 on page 28 need to be reorganized. Based on Tables 2 and 4, the DINA model produced a classification rate of .8 when the item quality was high for both blocked NPS and GNPS, as well as when the item quality was low and the test length was lengthy for the blocked NPS. While the A-CDM model yielded a classification rate of .8 with the high-quality item pool for the blocked GNPS and a high item quality with the longer test for the blocked NPS.**

**Reply:**

We appreciate the constructive suggestion that has allowed us to clarify this point, ensuring that our findings are accurately represented and understood.

These lines have been rewritten:

"*Our simulation studies demonstrated that under the DINA model, both blocked NPS and GNPS achieved a classification accuracy of 0.8 when item quality was high. Additionally, the blocked NPS model reached the satisfactory classification rate of 0.8 even when item quality was low, provided that the test length was sufficiently long. On the other hand, the A-CDM model showed that blocked GNPS achieved a satisfactory accuracy level of 0.8 with a high-quality item pool, and the blocked NPS reached the same level of accuracy when both the test length was extended and the item quality was high.*" (pp. 33-34)

6. **Before submitting the manuscript, please check for citations (e.g., introducing Q-matrix and attribute mastery pattern), grammatical errors, and coherence (e.g., missing notations for Equation 3, the simulation data generated by the DINA and A-CDM models but GNPS used the weights based on the DINA and DINO models on page 17).**

**Reply:**

Thank you for your thorough review and valuable feedback on our manuscript. We have made the following revisions to address each of your points:

1. **Citations**: We have carefully reviewed the manuscript and ensured that all necessary citations are included, particularly for the introduction of the Q-matrix and attribute mastery patterns. We have added the missing citations to provide proper attribution.
2. **Grammatical Errors**: We have conducted a comprehensive grammar check throughout the manuscript. All identified grammatical errors have been corrected to ensure that the text is clear, concise, and professional.
3. **Coherence**:
   - **Missing Notations for Equation 3 (Equation 4 in the revised manuscript)**: We have reviewed and updated Equation 4 to include all necessary notations.
   - **Clarification**: The erroneous description regarding GNPS has been corrected. A detailed explanation of the item selection strategy proposed in this study and used in the simulation study is provided in the section " The proposed blocked procedures."

We appreciate your meticulous attention to detail and the opportunity to enhance the quality of our manuscript. We believe that these revisions have significantly improved the clarity and coherence of our work.

Thank you once again for your constructive feedback. We are confident that our manuscript is now in a better position for publication.
